# Solid-Stemmed Wheat Does Not Affect Overwintering Mortality of the Wheat Stem Sawfly, *Cephus cinctus*

**DOI:** 10.1673/031.011.12901

**Published:** 2011-09-30

**Authors:** Héctor A. Cárcamo, Brian L. Beres, Carolyn E. Herle, Hugh McLean, Sean McGinne

**Affiliations:** Agriculture and Agri-Food Canada, Lethbridge Research Centre, 5403-1st Avenue South, Lethbridge, Alberta, Canada T1J 4B1

**Keywords:** cold-hardiness, novel solid wheat germplasm, supercooling, wheat stem sawfly, winter survivorship

## Abstract

The wheat stem sawfly, *Cephus cinctus* Norton (Hymenoptera: Cephidae), is a key pest of wheat in the northern Great Plains of North America. Host plant resistance in the form of solid-stemmed wheat cultivars is the main control strategy for *C. cinctus.* This study investigated the effect of novel and traditional solid wheat hosts on the overwintering mortality and cold-hardiness of *C. cinctus.* Field conditions from 2003–2005 showed that overwintering mortality in various wheat cultivars averaged 8% and was not related to the type of wheat cultivar. Similarly, supercooling points (-22° C) were not influenced by wheat host type. *C. cintus* are cold-hardy; up to 80% survive 10 days at -20° C and 10% survive 40 days. Its overwintering microhabitat near the crown area of the plant is well insulated for temperatures above -10° C and remains ∼ 20° C above ambient minima. These data suggest that winter mortality is a minor factor in the population dynamics of wheat stem sawfly, and despite clear detrimental effects on larval weight and adult fitness, solid-stemmed cultivars do not reduce the ability of larvae to survive winters.

## Introduction

Winter mortality can be a major factor in the population dynamics of many temperate, freeze-intolerant insects. To survive low temperatures, many insects have evolved the ability to super-cool well below zero (Bale 1987) and minimize their exposure to extreme ambient air temperatures by overwintering in sheltered habitats (Danks 1996). Pest forecasting and the implementation of IPM programs depend on such knowledge.

The wheat stem sawfly, *Cephus cinctus* Norton (Hymenoptera: Cephidae), is a serious and well-studied pest of wheat in the northern Great Plains of North America (Holmes 1979; Beres et al. 2011). In southern Canada, the short lived, non-feeding adults emerge from cereal stubble in mid to late June, then mate and lay eggs that hatch in about seven days (Ainslie 1920). Larvae feed inside the stem until kernel moisture content decreases to about 50% and, as day length shortens (Holmes 1975), migrate to the base where they girdle the stem about 25 mm above the crown to build an overwintering chamber and plug the cut stem with frass.

The cold-hardiness of *C. cinctus* was documented by Salt (1966a, 1966b) and Morrill (1993). Salt (1966a) reported a range of supercooling points between -22° C at a cooling rate of 1/28° C/min to -28° C at 14 °C/min. In a related study with bare larvae Salt reported that half—life (50% mortality) ranges from 1.2 sec at -30° C to 3.9 years at -16° C. At -20° C, their half-life was 7.4 days, demonstrating that insects could die at temperatures above their lethal supercooling points (Salt 1966b). Morrill et al. (1993) reported a range of half-lives from 4–8 hours at -20 °C and three hours at -22 °C. In the field, less than 8% winter mortality was found in undisturbed stubs, but over 90% mortality in stubs exposed to the surface by tillage in the fall (Morrill et al. 1993).

Host plant resistance is the primary pillar in the IPM program for *C. cinctus* (Holmes 1979). Genotypes with solid pith suffer, on average, at least 50% less damage than those with a hollow pith (DePauw et al. 1994). Additionally, several cultivars derived from the traditional S-615 Portuguese cultivar (Kemp 1934; Farstad 1940) have been registered in Canada and USA for commercial use. Recently, Clarke et al. (2005) registered a novel source of genetic resistance in a hexaploid wheat derived from a durum cultivar. Wheat genotypes with hollow piths produce heavier sawflies with higher reproductive potential than those that develop in hosts with solid pith (Cárcamo et al. 2005). Nevertheless, the effects of solid stem wheat on sawfly overwintering success are poorly understood. Morril et al. (1994) sampled material from field plot studies of winter wheat susceptible to sawfly and experimental solid-stemmed genotypes that were derived from the traditional S-615 source. These authors suggested that solid stem wheat increased overwintering mortality of sawfly, because they found few or no larvae alive in field overwintered solid stems and high numbers of healthy larvae in hollow stem material. However, the authors mentioned negligible levels of stem girdling by sawfly larvae in solid stem genotypes. Therefore, winter mortality was confounded by summer factors; larvae that fail to girdle the base of the stem to construct the overwintering chamber generally die during the summer (Holmes 1979).

Plant hosts have been shown to mediate winter mortality in a number of other insect-plant systems. Aphids that have contact with the host plant, particularly intact vs. excised leaves, have enhanced cold hardiness compared to those with no host contact (Butts et al. 1997). The nutritional quality of the host plant can also influence insect overwintering success. Liu et al. (2009) demonstrated that *Helicoverpa armigera* reared on kidney bean plants had higher winter mortality compared to those reared on cotton or artificial diet. A corresponding pattern in physiological activity of cryoprotectant compounds was noted by these authors and confirmed their earlier study (2007), which showed that *H. armigera* reared on superior hosts were better able to survive winters.

The objective of this study was to test the hypothesis that *C. cinctus* larvae that develop in lower quality, solid-stemmed hosts with traditional and novel solid pith traits have reduced cold-hardiness (higher supercooling points) and experience higher overwintering mortality under laboratory and field conditions compared to those that develop in conventional hollow-stemmed cultivars. The approach taken in our field study accounted for summer mortality by quantifying the survivorship of larvae that had successfully girdled wheat stems and constructed overwintering chambers.

## Materials and Methods

### Field study

The study was conducted from 2003 to 2006 at the Agriculture and Agri-Food Canada wheat stem sawfly research nursery (Beres et al. 2005), located 10 km west of Lethbridge, Alberta. The ten wheat genotypes (Table 1) included three hollow-stemmed susceptible hard red spring bread wheat cultivars (AC Barrie, AC Cadillac, McKenzie), two hollow-stemmed amber durum cultivars (AC Navigator, Kyle), two traditional solid-stemmed hard red spring bread wheat cultivars (AC Abbey, AC Eatonia), and three novel solid or partially solid-stemmed experimental synthetic hexaploid wheat lines (B9973B03&AC4AW, B9973B03&AG2AT and G9608B1-L12J11BF02). These novel germplasms containing the Golden Ball solid pith trait were derived by crossing a synthetic solid-stemmed wheat hexaploid (*Triticum turgidum* L. var. *durum*/*Aegilops squarrosa* L.) with a high-yielding hollow-stemmed hard red spring bread wheat (AC Elsa) (Clarke et al. 2005).

A self-propelled, six row precision cone seeder was used to sow plots to an area of 2 × 3 m, at a depth of 25–30 mm, and at a density of 200 seeds/m^2^. Seeding dates were 17 May 2003, 26 April 2004, and 2 May 2005. The herbicides Target (1.5 l/ha) mixed with Horizon (0.23 l/ha) were applied around mid to late June. Three sub-samples were collected in March 2004, 2005, and 2006 to measure winter mortality. Nine to 14 stubs (the base of a cut stem containing the overwintering larva) were dissected from each plot and around 10 live larvae were weighed and used to measure supercooling points. The larva inside each dissected stub was categorized as ‘live’ or fungal-, parasitoid-, or winter-killed. Our assumption of larva killed by fungi is supported by the work of Wenda-Piesik et al. (2009), who demonstrated that all fungi from *C.* *cinctus* cadavers dissected from stubs in their surveys in Montana were entomopathogenic members of a *Fusarium* spp complex. A similar complex was identified from a subsample of sawfly larval cadavers from our study site in another related study (Beres, unpublished data; Turkington, personal communication). Mortality by the parasitoid *Bracon cephi* was easily ascertained by counting either the parasitoid larval cocoon or its pinhead sized exit hole. A third biological mortality factor, likely caused by a predatory beetle, *Phyllobaenus dubius,* was also distinguished by its larger entry hole through the plug on the stub (Morrill et al. 2001). However, the incidence of these beetles was too infrequent for analysis. The stub outside diameter was measured 1 cm below the cut at three angles.

### Temperature data collection

Temperature was monitored 0.5 cm below the plug and at the crown level inside three cut stems and soil at the crown level adjacent to each of the three stems in a plot of the hollow stem cultivar AC Barrie, and an adjacent plot with the synthetic solid stem hexaploid G9608B1-L12J11BF02.

Temperature information was recorded with a CR10X-55 Datalogger (Campbell Scientific, www.campbellsci.com) using an AM25T 25-channel multiplexer and an SM4M-55 storage module (Campbell Scientific). A 0.8 mm hole was drilled at the target location in a cut stem. A 36-gauge copper-constantan (Type T) thermocouple (Omega Engineering, www.omega.com) was inserted into the stem and sealed with Mono Ultra Exterior waterproof silicone (Mono, www.monosealants.ca). 20-gauge type T wire soil probes (Omega Engineering) were buried at the crown level.

### Laboratory study

Two studies were conducted to determine the effects of solid- or hollow-stemmed wheat under a range of cold temperatures and periods of exposure. The first study, referred to as the ‘sublethal study’, consisted of a split-plot, repeated measures design with temperature (-5° C, -10° C, -20° C) as the main plot and wheat host type (AC Abbey = solid (DePauw et al. 2000), AC Cadillac = hollow (DePauw et al. 1998), G2AT = novel solid) as the subplot. The repeated measure was days of exposure (15 or 30 days). To quantify mortality upon completion of the exposure period, stubs were transferred to room temperature to record adult emergence. The response variable in this analysis was the proportion of survivors (out of 15 stubs), and the experimental unit was the sub sample of 15 stubs representing the ‘population’ of sawflies from that plant genotype. The second study, referred to as the ‘lethal study’, followed a similar split-plot design with temperature exposure as the main plot and cultivar as the subplot. Additionally, sawfly larva mortality was quantified in solid- and hollow-stemmed wheat cultivars (Table 1) at -20° C at 10, 20, 30, and 40 days. On the last year of the second lab study, only the two shorter exposure periods were tested, as several genotypes were needed to obtain enough samples of either host type. For the lethal study, mortality was measured after completion of the exposure period by storing the stubs at room temperature overnight before dissecting them. The cold temperature chamber treatment could not be replicated; therefore, the year and the year by temperature effects were considered random factors in both studies.

In both studies, plant stubs—cut by sawfly larva in the previous summer—were collected in late November or early December from the field plots and stored at 10° C. Before starting an experiment, each stub was buried in soil (12% moisture) to a depth of 2 cm above the crown in a 15 mL clear plastic vial with a screw-on pin-holed lid. The vials with the wheat hosts were then buried upright in soil in a large plastic tub (35 × 30 × 16 cm). The vial position was randomly assigned a treatment number to determine its exposure length, and the entire tub was randomly assigned to a temperature treatment.

### Supercooling points

In 2003 and 2004, the supercooling system consisted of a conical-shaped aluminum block lowered by a computer-controlled gear shaft into a liquid-nitrogen tank, modified from the design of Timbers and Danks (1970). The system had a computer with in-house programmed software to cool at a rate of 2° C/min and record temperature every 10 sec. T type 24 gauge thermocouples (Omega Engineering) were threaded through the lids of 2 mL screw-cap cryogenic vials. Four insects were weighed and fixed individually onto the thermocouples using lightweight polyester sewing thread (Gütermann, www.guetermann.com) and Pennlith grease (Pennzoil, www.pennzoil.com) for each run, which started at ∼20° C. The lowest temperature recorded before the release of the latent heat of fusion upon freezing was considered to be the supercooling point of each wheat stem sawfly. In 2005, the supercooling apparatus consisted of a thermoelectric AHP-301CP cold plate (Thermoelectric Cooling of America Corporation, www.thermoelectric.com). To prevent movement, each larva was encased in a small enclosure made of modeling clay. The thermocouple was placed lengthwise on the dorsal surface of the larvae and held in place with modeling clay. Thermocouple temperatures were recorded every 10 sec using a 21X Datalogger (Campbell Scientific). A Styrofoam insulated cover was placed on top of the cold plate. The supercooling system was moved to a +5° C walk-in cooler and the cooling run was initiated. However, the start temperature was still ∼20° C as the unit was not yet acclimatized to the cooler temperature. The cold plate was programmed for a cooling rate of 5° C/min, but linearity decreased below -10° C when the rate of cooling was closer to 1° C/min. After approximately 40 min, the run was completed.

**Table 1.**
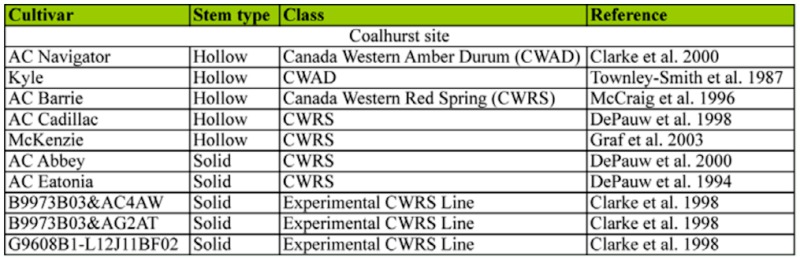
Cultivar, stem lumen type, and wheat class used in the 2003–2005 study of *Bracon cephi* and *Cephus cinctus* effects on wheat yield.

**Table 2.**
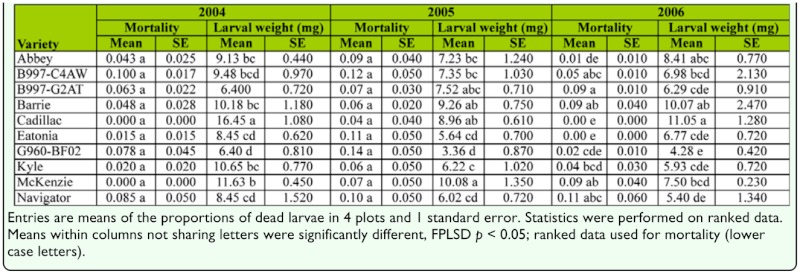
Proportion of sawfly larval mortality attributed to overwintering temperatures and body weights of live *Cephus cinctus* larva retrieved by dissecting stems.

### Data analysis

To estimate mortality attributed to winter temperature in all studies, the number of larvae killed by biotic factors such as *Bracon cephi*, fungi, or predators, or otherwise missing were subtracted from total mortality. The proportion of winter-killed larvae was arcsine transformed, but data are presented as proportions. In the field study, some wheat genotypes had zero mortality in one of the years (Table 2); therefore, the data were ranked transformed. For the field study, treatment effects were subjected to an ANOVA using the PROC Mixed procedure in SAS (SAS Institute 2002) with genotype considered a fixed effect, and block effects and block effects nested within main plots treated as random.

For both laboratory studies, years were used as replicates since only one cold chamber of each temperature (main plot) was available and wheat hosts were split into each chamber. Upon insect emergence or stem dissection, stems with parasitoids were counted and subtracted from total mortality, which was converted to proportion and arcsine transformed (square rooted first). An ANOVA was performed for a split plot design (Main Plot = temperature, subplot = wheat host) with repeated measures (days of exposure) using the PROC Mixed procedure of SAS version 9.2. The random effects were year and year × temperature, and the fixed effects were host, temperature, and days. For each response variable, separate models were run using CS, UN, or UN (1) as alternative covariance structures; the one with the lowest AICC value was selected for the final analysis, and the lowest level interaction with statistical significance (*p* < 0.05) or marginal significance (0.1 > *p* > 0.05) was selected for the error term and comparisons of means (LSD, *p* < 0.05). The analysis of the second lab experiment was similar except that temperature was not a factor; only host (fixed effect) with days of exposure as the repeated measure, and years and year × host as random factors. The second experiment resulted in an unbalanced design because the 30 and 40-day exposures could not be tested in 2006. Therefore, this year was excluded to assess all four exposure periods in 2004 and 2005. A second analysis was performed to include all three years but including only the 10 and 20 day exposures.

Supercooling points were analyzed as a fully randomized design using the PROC Mixed procedure in SAS® described above. Wheat host and days-of-exposure were fixed effects for larvae obtained from the above laboratory experiments; wheat host and time of collection (November or March) were fixed effects for the field study. The supercooling points were not compared for the field studies between the two growing seasons (2003 and 2004) because of differences in equipment used. Effects of wheat host were tested either keeping genotypes separate or by pooling them into solid or hollow-stemmed categories.

## Results

**Figure 1.**
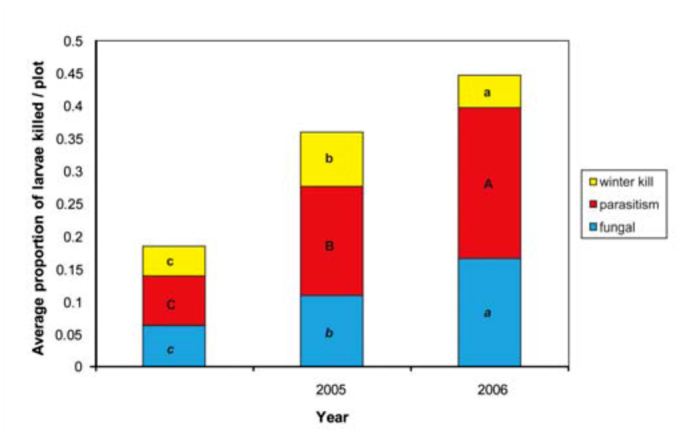
Average proportions of *Cephus cinctus* larva killed by abiotic (winter temperature) and biotic mortality factors (fungi, parasitoids) estimated from dissection of stems collected in March of 2004, 2005, and 2006. Entries are overall averages for 10 wheat genotypes and 4 replicates. High quality figures are available online.

Overwintering mortality in the field was low in all years and ranged from 0 to 13% with averages across all varieties around 5% in 2004 and 2006, and significantly higher (8%) in 2005 (Figure 1). The hollow-stemmed genotype AC Cadillac had the lowest mortality in all years; zero in 2004 and 2006, and the lowest of all cultivars in 2005 at 4% (Table 2). The highest mortality in 2004 and 2005 was observed in the solid genotypes B997-C4AW (10%) and G960-BF02 (13%), respectively. However, in 2006, the hollow-stemmed durum cultivar AC Navigator had the highest winter mortality at 10%.

The parasitoid *Bracon cephi* killed 7 to 23% of sawflies in the March field collections, which was slightly higher than mortality from fungal pathogens (Figure 1). Together, these biological factors inflicted higher mortality than winter conditions, and their contribution increased significantly in each successive year of the study. In 2006, ∼ 5% of stubs cut by sawflies (39 cases) had a large circular hole on the plug, suggesting predation by a beetle (Morrill et al. 2001; Beres et al. 2009); only one and 13 sawfly larvae were killed by this predator in 2004 and 2005, respectively.

**Figure 2.**
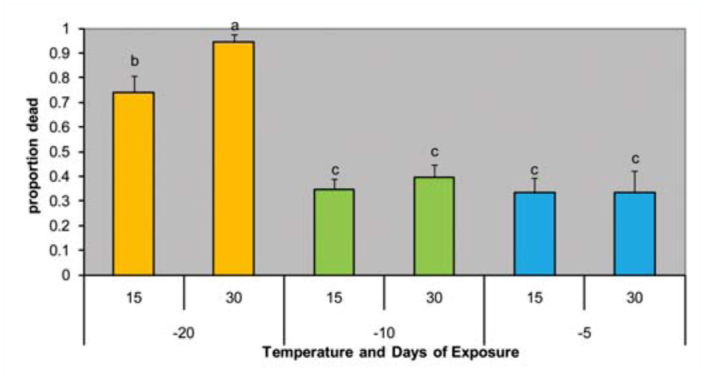
Mortality of *Cephus cinctus* larva at three sublethal cold temperatures (-5, -10, -20° C) after 15 or 30 days of exposure. Solid- and hollow-stemmed wheat genotypes had no effect and were pooled to estimate overall averages (N = 6). Means not sharing letters are significantly different, FPLSD < 0.05. High quality figures are available online.

In the laboratory experiment subjecting sawfly larvae to sub-lethal temperatures, the treatment at -20 °C killed significantly more larvae after 30 days of exposure than at 15 days (*p* < 0.05). Both of these treatments were significantly higher than the -5° C or -10° C treatments (Figure 2), though the larval host plant did not influence overwintering mortality. At the higher temperatures, mortality ranged from 34 to 40% and did not differ between days of exposure. Adult weight, wing length, and number of eggs per female were not affected by temperature or days of exposure (data not shown). Larval weights obtained in the overwintering field study (Table 2) demonstrated that hollow-stemmed genotypes produced consistently heavier larvae than one or more solid-stemmed genotypes, particularly compared to the novel solid genotype G960-BF02 (Table 2).

**Figure 3.**
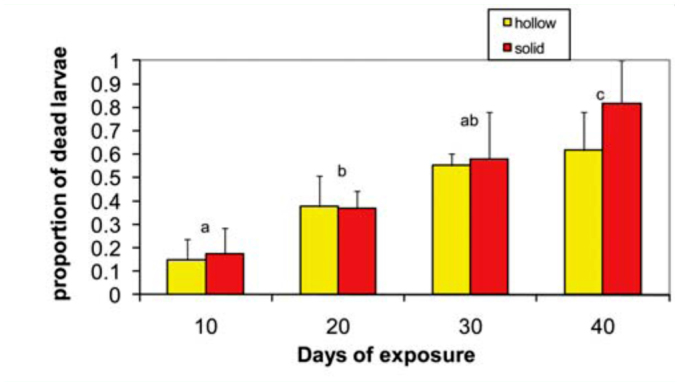
Effect of wheat host (solid or hollow) on mortality of *Cephus cinctus* larva in a cold chamber at -20° C after 10, 20, 30, or 40 days of exposure. Entries are means of three years for the 10 and 20 day exposure and two years for 30 and 40 day exposure. Means not sharing letters are significantly different, FPLSD <0.05. High quality figures are available online.

Dissections to assess larval mortality at -20° C in the two types of wheat revealed trends similar to those evident in the sub-lethal study. However, mortality was generally lower than that assessed using adult emergence as the response variable. After 30 days, larval mortality was around 60% instead of the 90% mortality measured when the insect was allowed to complete its life cycle to the adult stage. There was a significant increase in larval mortality at 40 days compared to 30 days (F_3,11_ = 14.59, *p* < 0.01; FPLSD *p* < 0.05, Figure 3). However, the mortality after 30 days (∼ 60%) did not differ significantly from the 10 and 20 day period, which averaged around 15 to 30%, respectively (FPLSD, *p* > 0.05). Although there was a numerical trend towards higher mortality in the solid- than in the hollow-stemmed host, the main host factor (individual cultivar or solid stemmed cultivars pooled) effect and its interaction with exposure were not significant (Figure 3).

**Figure 4.**
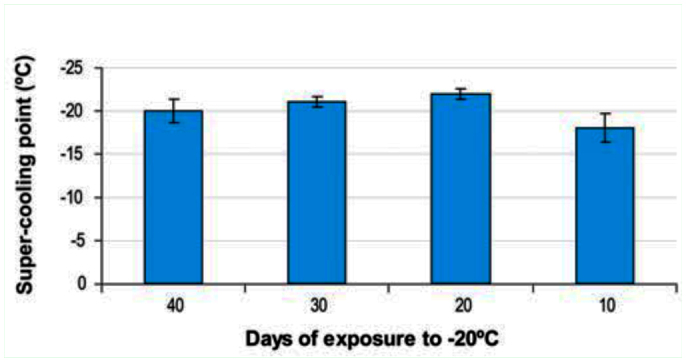
Supercooling points of *Cephus cinctus* larva after 10, 20, 30, and 40 days of exposure to -20° C. Wheat host type had no effect. Larval weight was used as covariate but was not significant; means not sharing letter were significantly different, FPLSD, *p* < 0.05. High quality figures are available online.

Supercooling points estimated from the liquid nitrogen apparatus ranged from -8 to 26° C with an overall average across all tests of -21° C. Wheat cultivar did not affect supercooling points in any of the experiments, nor did it interact significantly with field collection date (F_1,154_ = 2.58, *p* = 0.11). Larvae collected in March 2004 had slightly higher supercooling points (-21.4° C ± 0.18) than those collected in November 2003 (-22.2° C ± 0.18; F_1,154_ = 11.16, *p* < 0.01). Sawfly larvae that survived exposure to -20° C for 20, 30, or 40 days had slightly lower supercooling points than those that survived the 10 day period at this temperature (Figure 4). However, the overall ANOVA suggested no statistically significant differences.

**Figure 5.**
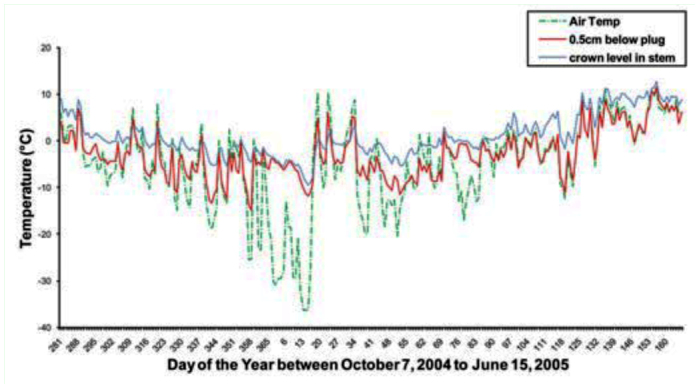
Temperatures at the field study site during 2004–2005 at 0.5 cm below top of plug in cut stub, inside the stem at the crown, and in both soil and air. Entries are means of three probes in solid and three probes in hollow-stemmed plants for each plant microhabitat. High quality figures are available online.

A sample of winter field temperatures in the various microhabitats is shown in Figure 5. Although air temperature dropped below -30° C, it remained above -10° C inside the cut stems at crown level and similar to soil temperature. The temperatures below the plug of the cut stub (∼ 2–4 cm above the crown) were a few degrees cooler than at crown level. In 2006, the cold temperature minima inside the cut stubs reached -20.7° C and -19.3° C in the hollow and solid cultivars, respectively. In 2005, the trend reversed and values of -13.3° C and -16.4° C were recorded.

## Discussion

Under the conditions of this study, relatively few sawfly larvae died from winter cold, compared to mortality from biotic factors. The average winter mortality across all wheat genotypes each year was less than 8%, which was the same value reported by Morrill et al. (1993) for field conditions in Montana, USA. In our study, more mature larvae in the cut stubs were killed by parasitoids (up to 23%) and fungi (up to 16%) than by winter temperatures. Host quality, in terms of stem type (hollow vs. solid), regardless of cultivar, had no effect on winter mortality. Although larvae that developed in resistant (solid) cultivars had consistently lower fresh weights than those that developed in susceptible (hollow) cultivars as observed in other studies (Cárcamo et al. 2005), this showed no negative effects on winter survival. Morrill et al. (1994) suggested that desiccation and freezing were responsible for the overwintering mortality of *C. cinctus* larvae inside uncut solid stem wheat in aboveground locations. In our study, this was clearly not the case; all larvae that failed to cut the stem, regardless of wheat cultivar, were found dead above ground prior to the onset of winter. This was likely also the case in the study by Morrill et al. (1994). In contrast to the findings of Liu et al. (2007, 2009) for *H. armiguera*, sawfly larval body size at overwintering did not affect survival, although there were clear effects of wheat genotype on larval weights. The lack of host effects in our study can be explained by the fact that temperatures inside the sawfly's microhabitat do not drop below lethal levels. Although air temperatures frequently dipped below -30° C in all years, the temperature inside stubs ∼ 0.5 cm below the cut remained above the sawfly's supercooling point of -22° C.

Supercooling points of *C.* *cinctus* were similar to those reported by Salt (1966a, 1966b) and Morrill et al. (1993), and showed no meaningful differences between larvae that developed in solid-stemmed hosts and the more susceptible hollow-stemmed wheat cultivars. Mortality of larvae at various sublethal cold temperatures and exposure periods also was not influenced by host type.

Cold-hardiness of *C. cinctus* larva differs between bare larvae and those protected in cut stubs (Salt 1966b). For bare larvae, Salt (1966b) and Morrill et al. (1993) quantified freezing time of 7.3 days and 4–8 hours, respectively, at -20° C. Salt (1966b) exposed larvae in stubs but did not report the freezing time. However, from the graph and regression equation provided, it can be estimated that 20–30% of larvae took from 30 to 70 days to freeze. Our results with protected larvae are in agreement with Salt (1966b), since we found that up to 63% and 38% (in hollow-stemmed wheat) of *C. cinctus* larvae were still alive after 15 and 40 days, respectively, at -20° C. It remains unknown why bare larvae freeze faster than protected larvae. Salt (1966b) discounted ice-nucleating moisture as a factor since this variable was controlled in his studies.

Temperatures in cut stubs rarely reached -20° C in our field study; they usually became buried as winter progressed, and were well insulated before the coldest temperatures were reached in January. About 0.5 cm below the plug, temperatures reached -20° C for less than two days, and at the crown level, to which sawfly hibernacula can reach, the temperature only reached -12° C. It is interesting that *C. cinctus* has such a high level of cold-hardiness given its protected microhabitat. In contrast, the cabbage seedpod weevils, that overwinter in field margins and tree shelters under leaf litter, have a supercooling point of -7° C, which is only 2–3 degrees below the winter temperatures in this microhabitat (Cárcamo et al. 2009). In contrast, *C. cinctus* has a supercooling point around 10° C below the minima of their microhabitat. Freezing intolerant insects usually have supercooling points that approximate the minimum temperatures of their overwintering microhabitats (Danks 1978), due to the physiological cost of cryoprotectant production (Liu et al. 2007). This suggests that winter temperature does not restrict the northern range of the sawfly; other factors may prevent this species from reaching pest status in more northern regions.

In summary, our study confirmed the cold-hardiness of the wheat stem sawfly and demonstrated that wheat cultivar (hollow/solid stemmed) has little influence on overwintering survivorship or supercooling points. Although larvae that developed in susceptible, hollow-stemmed wheat cultivars had higher body weights than those that developed in resistant, solid-stemmed wheat, they had similar cold hardiness and equally high probability (∼ 92%) of surviving the winter. Thus, the detrimental effects of resistant wheat cultivars on sawfly populations are attributable to their impact on summer survivorship, body mass, and adult reproductive potential as demonstrated in earlier studies, and are not attributable to any impact on overwintering survival.
